# Mitochondrial MUL1 E3 ubiquitin ligase regulates Hypoxia Inducible Factor (HIF-1α) and metabolic reprogramming by modulating the UBXN7 cofactor protein

**DOI:** 10.1038/s41598-020-58484-8

**Published:** 2020-01-31

**Authors:** Lucia Cilenti, Jacopo Di Gregorio, Camilla T. Ambivero, Thomas Andl, Ronglih Liao, Antonis S. Zervos

**Affiliations:** 1 Burnett School of Biomedical Sciences, University of Central Florida College of Medicine, 12722, Research Parkway Orlando, FL 32826 USA; 2 Stanford Cardiovascular Institute, Stanford University School of Medicine, 1651 Page Mill Road, Palo Alto, 94043 USA

**Keywords:** Ubiquitylation, Cancer metabolism

## Abstract

MUL1 is a multifunctional E3 ubiquitin ligase anchored in the outer mitochondrial membrane with its RING finger domain facing the cytoplasm. MUL1 participates in various biological pathways involved in apoptosis, mitochondrial dynamics, and innate immune response. The unique topology of MUL1 enables it to “sense” mitochondrial stress in the intermembrane mitochondrial space and convey these signals through the ubiquitination of specific cytoplasmic substrates. We have identified UBXN7, the cofactor protein of the CRL2^VHL^ ligase complex, as a specific substrate of MUL1 ligase. CRL2^VHL^ ligase complex regulates HIF-1α protein levels under aerobic (normoxia) or anaerobic (hypoxia) conditions. Inactivation of MUL1 ligase leads to accumulation of UBXN7, with concomitant increase in HIF-1α protein levels, reduction in oxidative phosphorylation, and increased glycolysis. We describe a novel pathway that originates in the mitochondria and operates upstream of the CRL2^VHL^ ligase complex. Furthermore, we delineate the mechanism by which the mitochondria, through MUL1 ligase, can inhibit the CRL2^VHL^ complex leading to high HIF-1α protein levels and a metabolic shift to glycolysis under normoxic conditions.

## Introduction

Eukaryotic cells are dependent on oxygen levels as well as the presence of functional mitochondria in order to efficiently generate ATP through oxidative phosphorylation (OXPHOS). Cells respond quickly to changes in oxygen levels by activating several signaling pathways that provide metabolic and adaptive mechanisms to the new environment. Hypoxia-inducible factor 1α (HIF-1α) is the primary transcriptional regulator of the cell response to low oxygen levels (hypoxia)^1–4^. Accumulation of HIF-1α protein and its translocation to the cell nucleus leads to the transcriptional activation of several hundred genes that carry a hypoxia response element (HRE) in their promoters^5,6^. This leads to HIF-1α-dependent reprogramming of cellular metabolism that shifts the ATP production from oxidative phosphorylation, that is limited under low oxygen levels, to glycolysis^7,8^. There is an important phenomenon associated with most cancer cells where glycolysis is predominantly used as the main source of ATP production, even under normal levels of oxygen (normoxia). This is known as the Warburg effect, originally described in 1923 and has since been extensively studied^9,10^. Accumulating evidence indicates that induced aerobic glycolysis is not only the hallmark of cancer, but it is also important in many cellular processes including embryogenesis, innate and adaptive immunity, type 2 diabetes, starvation, as well as cardiomyopathy^11–17^. The mechanism that potentially “bypasses” the tight regulation of cellular metabolism by the HIF-1α transcription factor is unknown. Under normoxia, HIF-1α is continuously synthesized and degraded in the cytosol through a highly regulated process. The oxygen sensor propyl hydroxylase 2 (PHD2) hydroxylates HIF-1α which then binds the von Hippel-Lindau (VHL) tumor suppressor protein and gets ubiquitinated by the CRL2^VHL^ ligase complex^18^.

We have uncovered a new pathway that regulates HIF-1α protein levels and involves the mitochondrial MUL1 E3 ubiquitin ligase. MUL1 (also known as Mulan, MAPL, GIDE, and HADES) is one of three mitochondrial E3 ubiquitin ligases, the other two being MARCH5 and RNF185^19–28^. Previous studies have shown MUL1 to be a major player in a number of pathways involved in apoptosis, mitophagy, and innate immune response^21,25,26,29–33^. MUL1 is able to modify specific substrates through SUMOylation, as well as K63- or K48-ubiquitination^20,21,34,35^. Our data show that MUL1, through K48-ubiquitination, directly regulates the level of UBXN7 protein, also known as UBX domain protein 7 (UBXD7)^36^. UBXN7 serves as a substrate binding adaptor and interacts with several proteins including HIF-1α, CUL2, as well as AAA + ATPase p97, also known as VCP (Valosin-containing protein)^36,37^. We identified lysine 14 (K14) and lysine 412 (K412) on the UBXN7 protein as the two major K48-ubiquitination sites for MUL1 ligase. Ubiquitination of UBXN7 targets the protein for proteasome degradation and inactivation of MUL1 leads to high levels of UBXN7 with concomitant increase in HIF-1α protein. The accumulation of HIF-1α is functional and is accompanied by activation of GLUT1, a known target of HIF-1α^38^. This deregulation of HIF-1α affects the metabolic state of cells with glycolysis becoming the predominant energy production pathway even during aerobic conditions. In summary, we describe a new mitochondrial-initiated pathway that interferes with the process of HIF-1α regulation and reprograms cellular metabolism to induce aerobic glycolysis.

## Results

### Isolation of UBXN7 as a substrate of mitochondrial MUL1 E3 ubiquitin ligase

**Figure 1:**
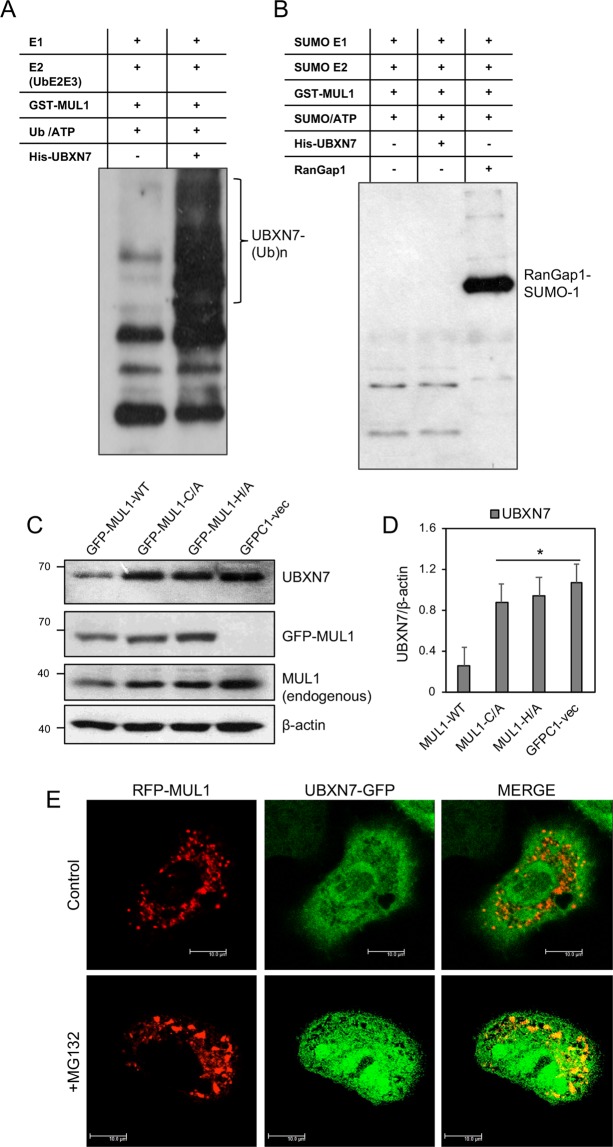
MUL1 ligase ubiquitinates UBXN7 *in vitro* and regulates its protein level *in vivo*. **(A)** UBXN7 can be ubiquitinated by MUL1 E3 ligase *in vitro*. His-UBXN7-Ub smear (hallmark of protein ubiquitination) was observed in the reaction that includes GST-MUL1 and His-UBXN7 substrate. **(B)** *In vitro* SUMOylation assay was also performed but no detectable SUMOylation of His-UBXN7 by MUL1 was observed. RanGap1protein was used as a generic substrate to verify the SUMOylation activity of the reaction. **(C)** Overexpression of the active GFP-MUL1 ligase, but not the inactive GFP-MUL1 mutants, affect the endogenous level of UBXN7 protein. HEK293 cells were transfected with GFP-MUL1, the inactive GFP-MUL1-C/A, GFP-MUL1-H/A or the GFPC1 vector as a control. Total cell lysates were collected after 24 hours and analyzed by SDS-PAGE and Western blotting using UBXN7 and MUL1 specific antibodies. β-actin was used to verify equal loading of proteins in each lane. **(D)** Graph represents densitometric analysis of the UBXN7 protein expression from (C) normalized against β-actin, *p < 0.002 *vs* GFP-MUL1-WT. **(E)** Co-localization of MUL1 and UBXN7 was studied in transfected cells. HeLa cells were plated on glass-cover slips and co-transfected with RFP-MUL1 and UBXN7-GFP. After 24 hours, one set of the transfected cells was treated with MG132 for 3 hours and another set was used as control. Cells were visualized using confocal microscopy. The first panel represents RFP-MUL1, the second represents UBXN7-GFP, and the last panel is a merged image showing co-localization of the two proteins as indicated by the yellow color. The bar represents 10 µm. All results shown are means ±S.D. of three independent experiments.

MUL1 can function as an E3 ligase and in association with E2 conjugation enzymes can ubiquitinate specific substrates both at K48 and K63^34,39–41^. In addition, MUL1 is able to SUMOylate various substrates and modulate their function^20,21,42^. We performed an unbiased proteomics assay to identify new substrates of MUL1 ligase. For this, a recombinant His-MUL1 (aa 259–352) polypeptide that represents the ring finger domain, which is responsible for the ligase activity, together with the full length His-UbE2E3 protein was used to profile 19,000 human poly-peptides for specific *in vitro* ubiquitination using a protein microarray (see Materials and Methods). Several potential substrates were isolated and one of them was the human UBXN7 protein. To further verify the ubiquitination of UBXN7 by MUL1, we produced recombinant full-length MUL1 (aa 1-352) as a fusion to Glutathione S-Transferase (GST) (GST-MUL1), a His-tagged full length UBXN7 protein (His-UBXN7), as well as His-tagged conjugation enzyme (His-UbE2E3). GST-MUL1 ubiquitinates the His-UBXN7 but it is unable to SUMOylate it (Fig. 1A,B). We investigated if K48-linked polyubiquitination of UBXN7 is involved, which would target the protein for proteasomal degradation, therefore we transfected HEK293 cells with a vector that expresses the full length MUL1 or two different mutants of MUL1 where an amino acid substitution abolishes its ligase activity^19^. Figure 1C shows overexpression of the wild type MUL1 substantially decreases the endogenous level of UBXN7 as compared to cells transfected with the GFPC1 vector alone (Fig. 1D). Furthermore, the decrease in UBXN7 was not seen with the ligase inactive mutants, GFP-MUL1-C/A or GFP-MUL-H/A (Fig. 1D). This suggests that the ability of MUL1 to function as an E3 ligase is necessary and essential for the degradation and removal of UBXN7 protein. MUL1 is predominantly found in the mitochondria, embedded in the outer mitochondrial membrane^19^. UBXN7 is a cofactor protein of the CRL2^VHL^ complex and is reported to be present both in the cytoplasm as well as the nucleus^36,43,44^. We co-expressed RFP-MUL1 and UBXN7**-**GFP in HeLa cells and the subcellular localization of these proteins was monitored under normal conditions or in the presence of the proteasome inhibitor MG132. Figure 1E shows the punctate staining of RFP-MUL1 that is characteristic of mitochondrial distribution, whereas UBXN7-GFP shows expression throughout the cell. When the two images were merged, they show extensive co-localization between the two proteins in the mitochondria. Cells treated with the MG132 inhibitor show the same subcellular distribution of the two proteins and increased intensity in the co-localization of UBXN7-GFP with RFP-MUL1 due to the added stability and accumulation of the two proteins.

### Mapping the UBXN7 ubiquitination sites on conserved residues K14 and K412

**Figure 2:**
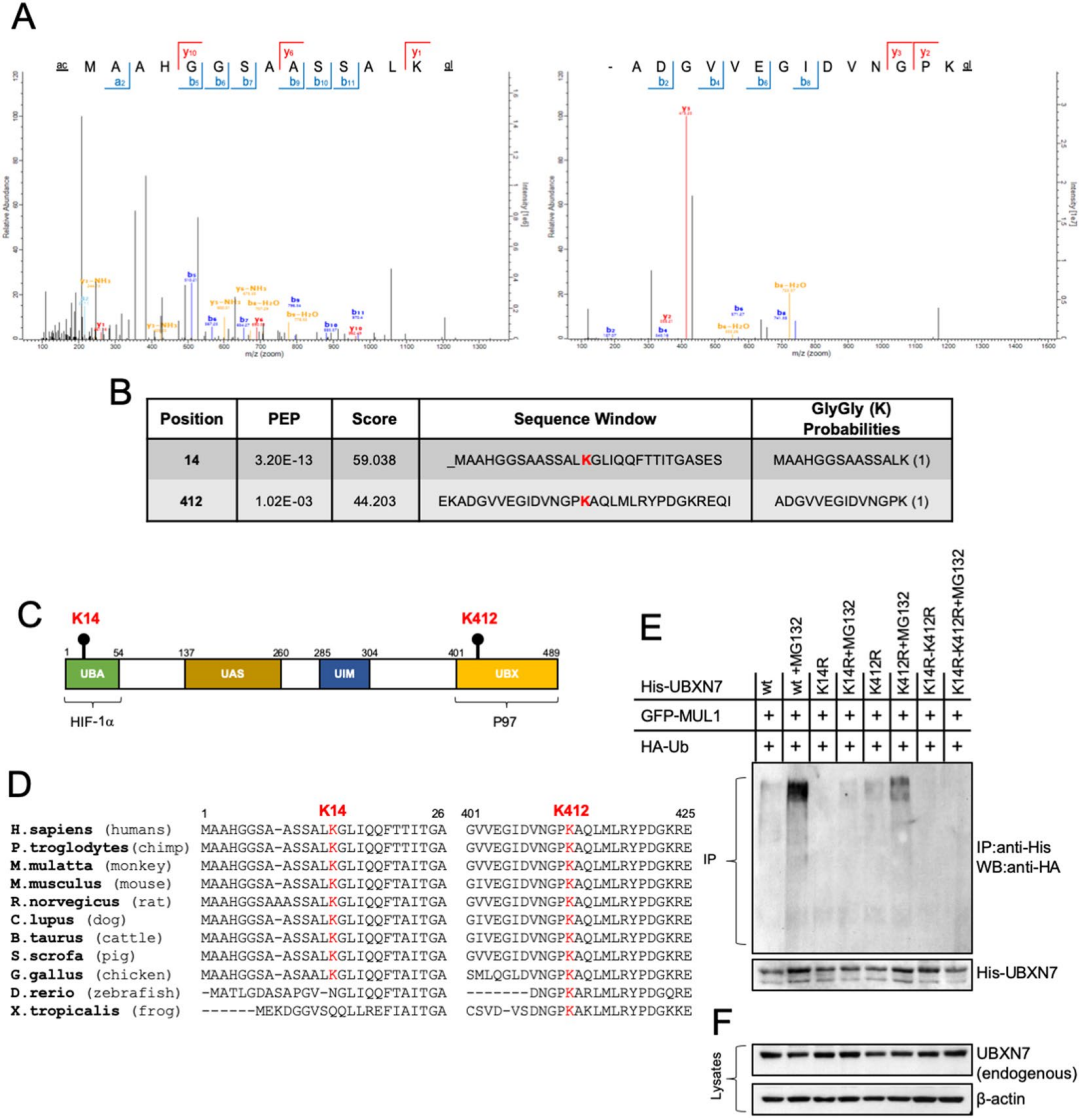
Identification of the ubiquitination sites on UBXN7. **(A)** Mass spectrometry data of enzymatic digested peptides from His-UBNX7 was acquired on Nanoflow UPLC and analyzed using MaxQuant. **(B)** The two ubiquitination sites of His-UBXN7 on K14 and K412 are highlighted and shown on the peptide sequence. Trypsin digestion of ubiquitin conjugates generates a diGly tag that is formed at the ubiquitinated lysine residue. **(C)** Schematic diagram of UBXN7 protein showing the different domains and indicating the respective location of the two ubiquitination sites. The K14 site is located in the UBA domain that is responsible for interaction domain with HIF-1α, whereas the K412 site is in the UBX domain which interacts with AAA + ATPase p97. **(D)** Amino acid sequence alignment of the two ubiquitin-modified lysine residues to highlight conservation among species. **(E)** To confirm UBXN7 ubiquitination at the specific lysines, both K14 and K412 were replaced with arginine (R). HEK293 cells were transfected in duplicate with the indicated plasmids: GFP-MUL1, pcDNA-HA-Ub, and His-UBXN7 wild type (wt), single mutants His-UBXN7-K14R or -K412R and double mutants His-UBXN7-K14R-K412R. Cell lysates from control and MG132 treated plates were immunoprecipitated using anti-His antibodies, the precipitate resolved by SDS-PAGE and Western blot analysis using HA antibodies. Lower panel shows His-UBXN7 expression. **(F)** Western blot analysis of the transfected cells used in (E) to verify overexpression of the different His-UBXN7 constructs does not affect the endogenous level of UBXN7 protein.

To evaluate the ubiquitination of UBXN7 and map the amino acid residues involved, we transfected HEK293 cells with pcDNA-(His)_6_-UBXN7 vector to overexpress His-UBXN7 in the presence of MG132 in order to enrich for K48-linked polyubiquitinated proteins. His-UBXN7 was purified and through mass-spectrometric analysis two ubiquitination sites, K14 and K412, were identified (Figs. 2A,B). K14 is located in the UBA domain known to be involved in the interaction with HIF-1α whereas K412 is located in the UBX domain that interacts with the AAA + ATPase p97 (Fig. 2C)^36,44^. Both lysine residues are highly conserved in the UBXN7 protein across various species (Fig. 2D). To validate the authenticity of the ubiquitination sites, we created individual point mutants as well as a double mutant where both lysine residues at position 14 and 412 were replaced with arginine. These constructs were transfected into HEK293 cells together with GFP-MUL1 and HA-Ub. Half of the cells were treated with MG132 to block the degradation of the K48-ubiquitinated proteins. His-tagged UBXN7 and mutant proteins were isolated on a Ni-NTA resin column and their ubiquitination profile was monitored by SDS-PAGE and Western blot analysis (Fig. 2E). UBXN7-K14R mutant has a substantial reduction in the overall ubiquitination compared to the wild type protein. UBXN7-K412R shows little difference in the overall ubiquitination as compared to the wild type protein. There was no detectable ubiquitination in the double mutant UBXN7-K14R-K412R. These data suggest that K14 is likely the major ubiquitination site on the UBXN7 protein. Overexpression of the various UBXN7 mutants (UBXN7-K14R, UBXN7-K412R, and UBXN7-K14R-K412R) proteins had no effect on the endogenous UBXN7 protein level (Fig. 2F).

### UBXN7 protein level controls HIF-1α accumulation and activity

**Figure 3:**
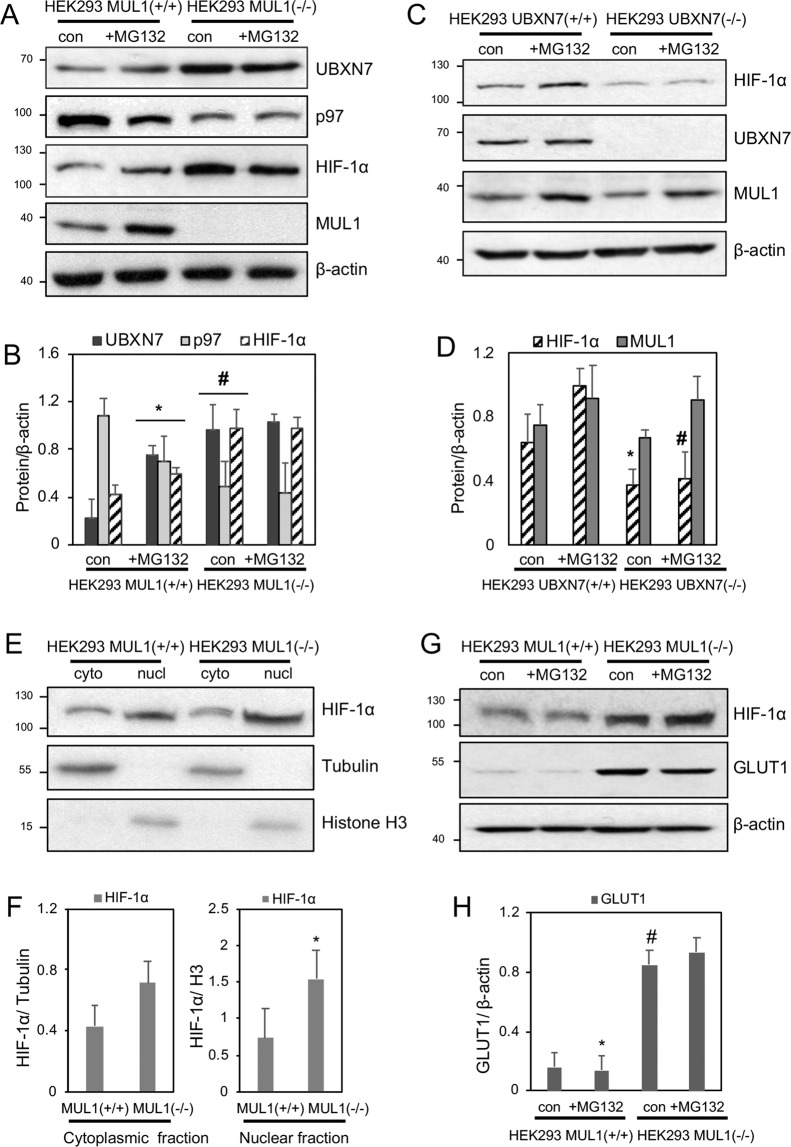
Correlation between UBXN7 protein level with HIF-1α accumulation and activity. HEK293 MUL1(+/+) and HEK293 MUL1(−/−) cells were treated with MG132 for 4 hours, whole cell extracts were prepared, and the expression of the indicated proteins was monitored by SDS-PAGE and Western blot analysis. **(A)** Expression of UBXN7, p97, HIF-1α and MUL1 proteins. β-actin antibody was used to verify equal loading in each lane. **(B)** Graph represents densitometric analysis of the protein expression from (A) normalized against β-actin. *p < 0.003 and #*p* < 0.04 *vs* HEK293 MUL1(+/+) control. **(C)** HEK293 UBXN7(+/+) and HEK293 UBXN7(−/−) cells were treated with MG132 for 4 hours. The expression of HIF-1α, MUL1, and UBXN7 was monitored by SDS-PAGE and Western blot analysis using specific antibodies. **(D)** Graph represents densitometric analysis of the protein expression from (C) normalized against β-actin. *p < 0.007 *vs* HEK293 UBXN7(+/+) control and #*p* < 0.0009 *vs* HEK293 UBXN7(+/+) + MG132. Results shown are means ± S.D. of three independent experiments. **(E)** Cytosolic and nuclear fractions from HEK293 MUL1(+/+) and HEK293 MUL1(−/−) cells. Expression of HIF-1α was monitored as described above, tubulin and histone H3 antibodies were also used to verify any potential cross-contamination between the two fractions. **(F)** Protein levels of HIF-1α normalized against tubulin and histone H3. *p < 0.03 *vs* HEK293 MUL1(+/+) nuclear fraction. **(G)** Accumulation of HIF-1α in HEK293 MUL1(−/−) cells leads to increased expression of its target GLUT1 protein. **(H)** Graph represents densitometric analysis of the GLUT1 protein expression from (**G**) normalized against β-actin, *p < 0.0001 HEK293 MUL1(+/+) control and #*p* < 0.0001 *vs* HEK293 MUL1(+/+) + MG132. Results shown in this figure are means ± S.D. of three independent experiments.

We used HEK293 T-REx Flp-In HEK293 MUL1(+/+) and HEK293 MUL1(−/−) cells described in^41^ and kindly provided by Dr. Janos Steffen and Dr. Carla Koehler (Department of Biological Chemistry, David Geffen School of Medicine, Los Angeles, CA). Figure 3A,B show that HEK293 MUL1(−/−) cells express increased levels of UBXN7 and are not affected by MG132, suggesting that MUL1 is the ligase responsible for UBXN7 ubiquitination. The HEK293 MUL1(+/+) cells express lower levels of UBXN7 protein that is increased in the presence of MG132, suggesting ubiquitination by MUL1 is involved in the regulation of UBXN7 protein level. UBXN7 is the adaptor protein of the CRL2^VHL^ ligase complex that is involved in the regulation of HIF-1α^36,45^. UBXN7 directly interacts with both HIF-1α as well as AAA + ATPase p97^36,43^. Furthermore, previous studies have shown that overexpression of the UBXN7 protein can inhibit the normal function of the CRL2^VHL^ complex leading to accumulation of HIF-1α^36,43^. HEK293 MUL1(−/−) cells showed high levels of UBXN7 as well as accumulation of HIF-1α but reduced levels of AAA + ATPase p97 protein. The blot was also re-probed with MUL1 antibodies to verify lack of expression in the HEK293 MUL1(−/−) cells. As expected, MUL1 expression is completely absent in HEK293 MUL1(−/−) cells; HEK293 MUL1(+/+) cells show robust expression of MUL1 that increases in the presence of MG132, due to partial regulation of the MUL1 protein through auto-ubiquitination^25,40^. To further investigate the correlation between the UBXN7 and HIF-1α protein levels, we used CRISP/Cas9 gene editing to create HEK293 UBXN7(−/−) cells. Figure 3C,D show HEK293 UBXN7(−/−) cells express substantially lower levels of HIF-1α protein when compared to HEK293 UBXN7(+/+) cells. These results provide additional support that it is the UBXN7 level alone that determines the accumulation of HIF-1α in the cells. In addition, there was no detectable change in the MUL1 level in the absence of UBXN7 expression. To verify that the increase in the HIF-1α in HEK293 MUL1(−/−) cells is relevant to the function of this protein, we monitored the subcellular distribution of HIF-1α between the cytoplasm and the nucleus. Although the regulation of HIF-1α protein takes place in the cytoplasm, translocation to the nucleus is necessary in order for the protein to function as a transcription factor. Figure 3E,F show a Western blot analysis of the subcellular distribution of HIF-1α in HEK293 MUL1(+/+) and HEK293 MUL1(−/−) cells. Tubulin and histone H3 antibodies were used on this blot to monitor the success of the cell fractionation. HIF-1α is present in both compartments and there is substantial accumulation in the nucleus of the HEK293 MUL1(−/−) cells. These results suggest that increased levels of UBXN7 in HEK293 MUL1(−/−) cells lead to nuclear accumulation of HIF-1α. To further confirm the effect of MUL1 ligase on UBXN7 is post-translational, we performed qRT-PCR to monitor the mRNA levels of UBXN7 in HEK293 MUL1(+/+) and HEK293 MUL1(−/−) cells. There is no significant change in UBXN7 mRNA in the presence or absence of MUL1 (Supplementary Fig. S1). We also monitored the mRNA level of VEGF-A, a known target of HIF-1α^17^. VEGF-A shows significant induction of expression in the absence of MUL1 (Supplementary Fig. S1). This is consistent with the accumulation of HIF-1α in the HEK293 MUL1(−/−) cells. The expression of GLUT1 protein, another target of the HIF-1α transcriptional factor, was also investigated in HEK293 MUL1(+/+) and HEK293 MUL1(−/−) cells. GLUT1 protein level correlates with the accumulation of HIF-1α and it is substantially higher in HEK293 MUL1(−/−) cells. In additions, treatment of HEK293 MUL1(−/−) cells with MG132 has no effect on the protein level of GLUT1, suggesting ubiquitination and proteasome degradation is not involved in its accumulation (Figs. 3G,H).

### Expression of various components of the CRL2^VHL^ ligase complex in MUL1 null cells

**Figure 4:**
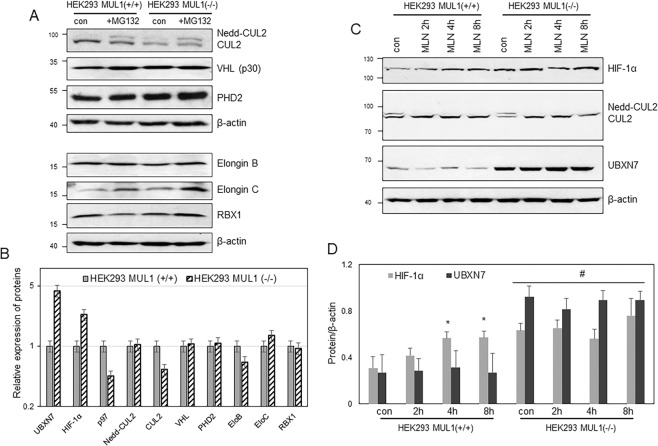
Expression of the various components of the CRL2^VHL^ complex in the presence or absence of MUL1 protein. **(A)** The differential expression of CUL2, VHL, PHD2, Elongin B, Elongin C and RBX1 proteins in HEK293 MUL1(+/+) and HEK293 MUL1(−/−) cells was monitored by SDS-PAGE and Western blot analysis. β-actin antibody was used to verify equal loading in each lane. **(B)** Graph summarizing the differential modulation of the various components of the CRL2^VHL^ complex between the HEK293 MUL1(+/+) and HEK293 MUL1(−/−) cells using densitometric analysis of the protein expression data from Figs. 3A and 4A. The scale of the graph is presented as log_5_. **(C)** HEK293 MUL1(+/+) and HEK293 MUL1(−/−) cells were treated with the neddylation inhibitor MLN4924 (1 μM) for various time point (2, 4 and 8 hours). The expression of UBXN7, HIF-1α, CUL2 as well as neddylated-CUL2 was monitored. **(D)** Graph represents densitometric analysis of the UBXN7 and HIF-1α protein expression from (**C**) normalized against β-actin. *p < 0.03 *vs* HEK293 MUL1(+/+) control and ^#^*p* < 0.001 *vs* HEK293 MUL1(+/+). Results shown are means ± S.D. of three independent experiments.

HIF-1α protein levels are low during normoxia, where the oxygen-sensitive PHD2 hydroxylates HIF-1α on two propyl residues leading to its interaction with the VHL that is part of the CRL2^VHL^ ligase complex^18,46^. This complex contains a number of other components including CUL2, Elongin B, Elongin C, and RBX1 as the core subunits, as well as the UBXN7 cofactor protein and the AAA + ATPase p97 hexamer. The UBXN7 adaptor protein interacts with ubiquitinated HIF-1α via its UBA domain, with neddylated CUL2 via its UIM domain, and with AAA + ATPase p97 via its UBX domain^36,43^. Assembly of the whole complex is necessary for the ubiquitination, extraction, and HIF-1α’s subsequent degradation by the proteasome. We investigated whether the high levels of the adaptor UBXN7 might affect the protein levels of other components of the CLR2^VHL^ complex. Extracts were prepared from HEK293 MUL1(+/+) or HEK293 MUL1(−/−) cells and Western blot analysis was used to monitor the various proteins of the CLR2^VHL^ complex. In addition, some cells were treated with MG132 to investigate if proteasomal degradation might be involved in their potential regulation. CUL2 protein level decreases in the HEK293 MUL1(−/−) cells but its neddylated form slightly increases and appears more prominent in the presence of MG132 (Fig. 4A). There is no significant change in the protein levels of VHL (p30), PHD2 or RBX1, while Elongin B decreases, and Elongin C slightly increases. Figure 4B compares the relative protein levels of the various components of the CLR2^VHL^ complex between HEK293 MUL1(+/+) and HEK293 MUL1(−/−) cells using the data from Figs. 3A and 4A. Since UBXN7 interacts with neddylated CUL2, we investigated if the upregulation of HIF-1α that accompanies UBXN7 accumulation may involve deregulation of neddylation. We used a specific inhibitor of neddylation, MLN4924, and monitored its effect on the HIF-1α protein level at various time intervals^43,45,47^. Figures 4C,D show that in HEK293 MUL1(+/+) cells, MLN4924 treatment leads to accumulation of HIF-1α protein over time as previously described^48,49^. To the contrary, the same MLN4924 treatment has no effect on the higher HIF-1α protein level present in the HEK293 MUL1(−/−) cells. There is no detectable change in the level of UBXN7 with MLN4924 treatment. These experiments suggest that the CLR2^VHL^ complex is inactive in HEK293 MUL1(−/−) cells and that UBXN7 interaction with neddylated CUL2 is not involved in the HIF-1α accumulation seen in these cells.

### The role of MUL1 in the regulation of UBXN7, and HIF-1α proteins as well as apoptosis during hypoxia

**Figure 5:**
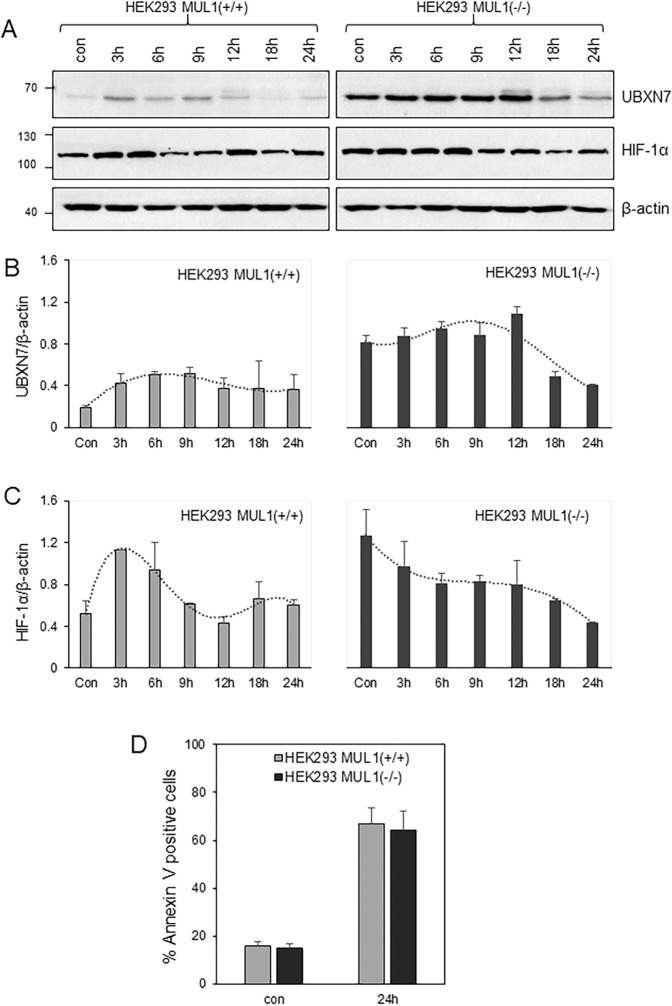
Hypoxic regulation of HIF-1α in the absence of MUL1. **(A)** Expression of UBXN7 and HIF-1α proteins in HEK293 MUL1(+/+) and HEK293 MUL1(−/−) cells that experience hypoxia (1% O_2_, 5% CO_2_, and 94% N_2_) or normoxia (5% CO_2_) at 37 °C for various time periods. Cell lysates were resolved by SDS-PAGE and the expression of UBXN7 and HIF-1α proteins were monitored by Western blot using specific antibodies. Graphs represent densitometric analysis of the UBXN7 **(B)** and HIF-1α **(C)** protein expression, from (**A**) normalized against β-actin. **(D)** The degree of apoptosis in control (normoxic) and 24 hour of hypoxia cell populations was examined by Annexin V staining and flow cytometry. Results shown are means ±S.D. of three independent experiments.

Our results clearly show that inactivation of MUL1 leads to accumulation of UBXN7 and HIF-1α proteins during normoxia. Next, we investigated whether the regulation of HIF-1α under conditions of hypoxia is also affected. HEK293 MUL1(+/+) and HEK293 MUL1(−/−) cells were placed in a hypoxia chamber for various time periods, whole cell extracts were prepared and the expression of HIF-1α as well as UBXN7 were monitored by Western blot analysis. In addition, the degree of apoptosis in the hypoxic cell population was monitored. The protein levels of UBXN7 and HIF-1α are much higher in the HEK293 MUL1(−/−) compared to the HEK293 MUL1(+/+) cells (Fig. 5A,B). In HEK293 MUL1(+/+) cells the UBXN7 and HIF-1α levels increase with hypoxia as previously described, and there seems to be a biphasic regulation of HIF-1α after 9 or 24 hours of incubation (Fig. 5C), which is not seen with UBXN7 (Fig. 5B). These results suggest that the HEK293 MUL1(−/−) cells have lost the regulation of HIF-1α during hypoxia, and they express the highest possible level of HIF-1α protein both during normoxia (control cells) and hypoxia. Although apoptosis in the cell population increased after 24 hours of hypoxia there was no significant difference between HEK293 MUL1(+/+) and HEK293 MUL1(−/−) cells (Fig. 5D), suggesting that the deregulation of HIF-1α is not involved in cell survival but might affect another function of HIF-1α, such as the regulation of metabolism.

### Mitochondrial respiration, ATP production, and glycolysis in the presence or absence of MUL1

**Figure 6:**
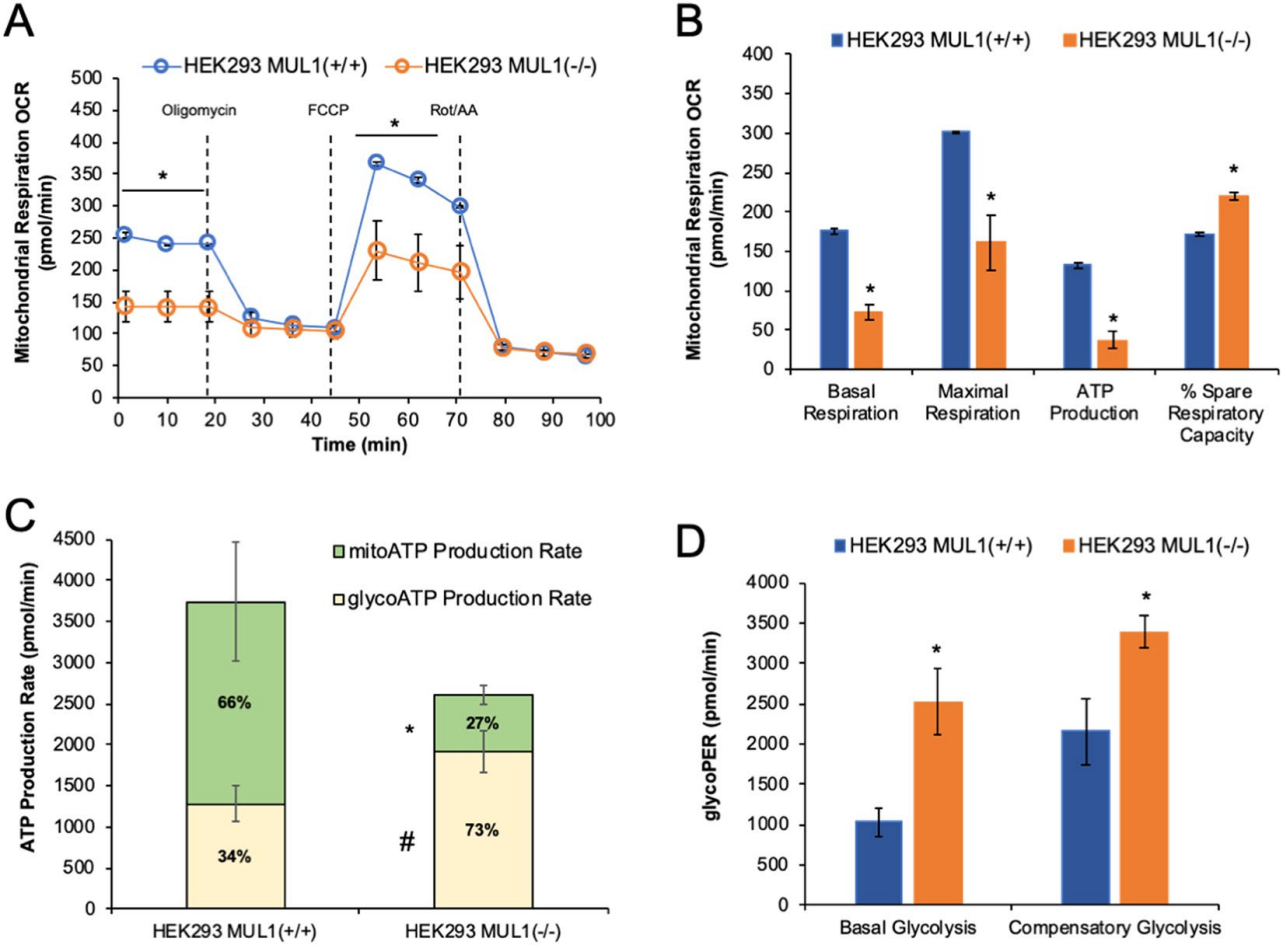
Mitochondrial respiration, ATP, and glycolysis rates in HEK293 MUL1(+/+) and HEK293 MUL1(−/−) cells. (**A)** Mitochondrial respiration in HEK293 MUL1(+/+) and HEK293 MUL1(−/−) cells was monitored using the Mitochondrial Stress Test. Oxygen consumption rate (OCR) and extracellular acidification rate (ECAR) were measured using the Seahorse XF^e^24 Extracellular Flux Analyzer. Trace shows representative data from one of three experiments. Results are expressed as mean ± SEM. *p < 0.01 *vs* HEK293 MUL1(−/−). **(B)** Quantification of the mitochondrial respiration data for basal respiration, maximal respiration, ATP production and spare respiratory capacity obtained from three independent experiments. *p < 0.01 *vs* HEK293 MUL1(+/+). **(C)** The ATP production rate in HEK293 MUL1(+/+) and HEK293 MUL1(−/−) cells was measured using the real-time ATP Rate Assay. Mitochondrial and glycolytic ATP production in HEK293 MUL1(+/+) and HEK293 MUL1(−/−) cells was determined. In HEK293 MUL1(−/−) cells, there is an increase in ATP made by glycolysis (glycoATP) compared to HEK293 MUL1(+/+) cells, indicating a shift from oxidative phosphorylation (mitoATP) to glycolysis. Data from three separate experiments are presented as means ± SEM. ^#^*p* < 0.05 *vs* glycoATP, *p < 0.05 *vs* mitoATP in HEK293 MUL1(+/+). **(D)** The Glycolytic Rate Assay was used to measure glycolysis as well as compensatory glycolysis of HEK293 MUL1(+/+) and HEK293 MUL1(−/−) cells. The Glycolytic Proton Efflux Rate (glycoPER) was converted from OCR and ECAR data through Seahorse XF^e^24 Glycolytic Rate Assay Report. Data from three separate experiments are presented as means ± S.E.M. *p < 0.04 *vs* HEK293 MUL1(+/+).

The Mito Stress assay was performed using the Seahorse XF^e^24 analyzer. Figure 6A is a trace of the mitochondrial respiration (oxygen consumption rate, OCR) results of HEK293 MUL1(+/+) and HEK293 MUL1(−/−) cells treated with specific inhibitors. Quantification of the results shows a significant decrease in the basal and maximal respiration in HEK293 MUL1(−/−) compared to HEK293 MUL1(+/+) cells, an inverse correlation in the spare respiratory capacity, as well as a decrease in ATP production via OXPHOS (Fig. 6B). The ATP Rate assay was also used to monitor mitochondrial respiration and glycolysis in HEK293 MUL1(+/+) and HEK293 MUL1(−/−) cells. In HEK293 MUL1(−/−) cells, the ATP rate is significantly reduced, but 73% of the ATP is now derived from glycolysis compared to 34% in HEK293 MUL1(+/+) cells, indicating a shift from OXPHOS to glycolysis (Fig. 6C). To further verify these data, we performed a Glycolytic Rate assay to monitor glycolysis as well as compensatory glycolysis of HEK293 MUL1(+/+) and HEK293 MUL1(−/−) cells. Figure 6D shows the Glycolytic Proton Efflux Rate (glycoPER) results converted from OCR and extracellular acidification rate (ECAR) data. Both the basal and compensatory glycolysis are significantly higher in HEK293 MUL1(−/−) compared to HEK293 MUL1(+/+) cells.

## Discussion

**Figure 7:**
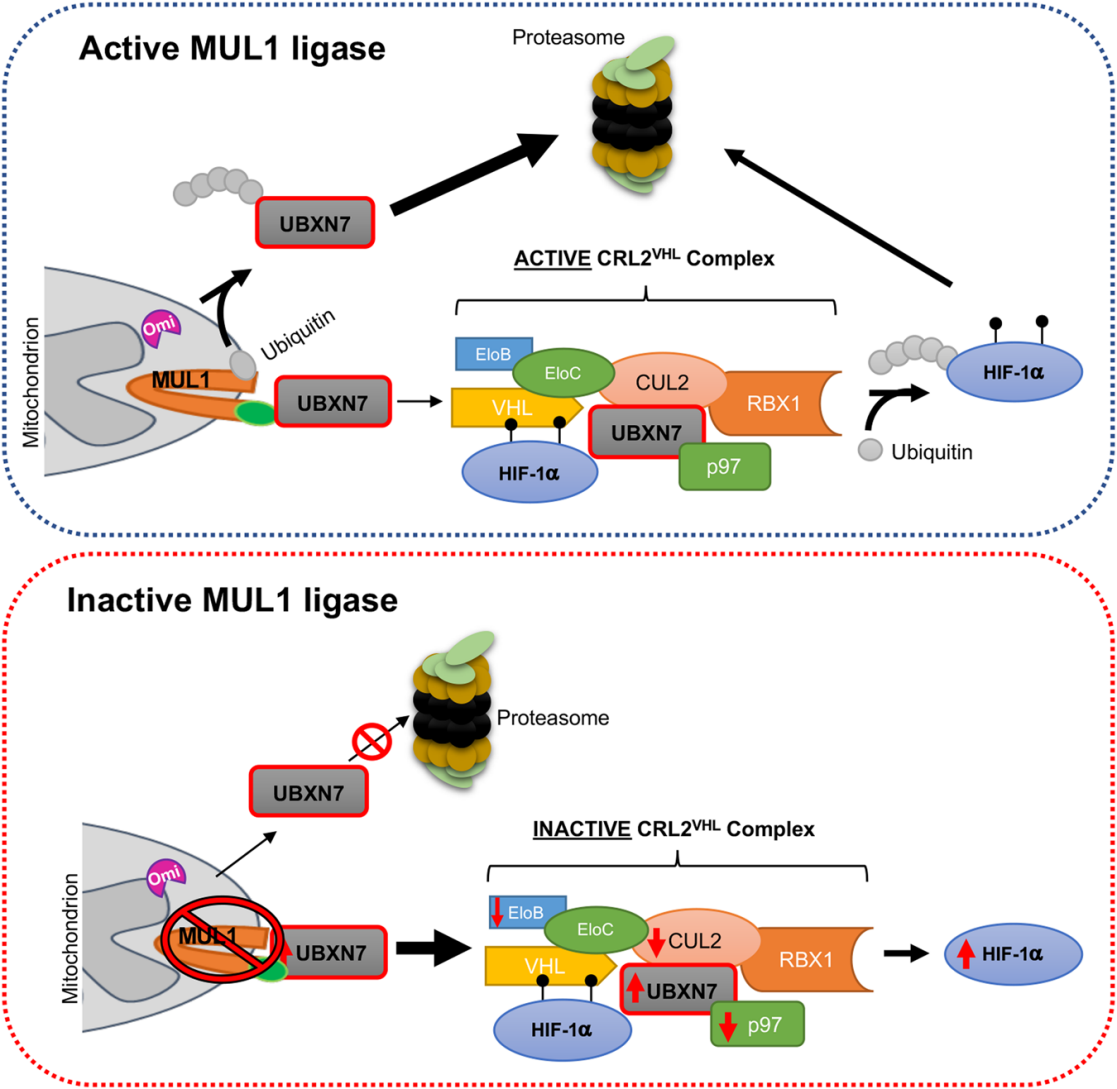
Schematic diagram of the proposed new pathway that operates upstream of the CRL2^VHL^ complex and involves the UBXN7 cofactor protein and its regulation by mitochondrial MUL1 E3 ligase. MUL1 ligase, through constant K48-linked polyubiquitination, maintains a steady low level of UBXN7 protein that is able to act as cofactor in assembling the active CRL2^VHL^ complex necessary for the regulation of HIF-1α during normoxia. When MUL1 becomes inactive or its activity is compromised, it leads to high levels of UBXN7 protein that function as an inhibitor of the CRL2^VHL^ complex. Without an active CRL2^VHL^ complex HIF-1α protein accumulates and drives glycolysis under normoxia. MUL1 protein levels are regulated by K48-autoubiquitination as well as by the action of the mitochondrial Omi/HtrA2 protease.

The intimate association between the amount of oxygen available in the mitochondria and the levels of cytoplasmic HIF-1α protein suggest that the mitochondria can directly regulate HIF-1α levels. The principal and best characterized regulatory mechanism of the HIF-1α protein level is through the CRL2^VHL^ E3 ubiquitin ligase complex^18,50^. During normoxia, the oxygen-sensitive PHD2 hydroxylates HIF-1α on two residues, Pro-402 and Pro-564^51^. Once hydroxylated, HIF-1α interacts with the VHL tumor suppressor protein, which is part of the CRL2^VHL^ complex, gets ubiquitinated and is rapidly degraded by the proteasome (Fig. 7). During hypoxia, hydroxylation of HIF-1α by PHD2 is inhibited which in-turn blocks VHL binding and ubiquitination leading to HIF-1α accumulation. Inhibition of PHD2 is reported to occur by the action of reactive oxygen species (ROS), auto-oxidation, intracellular cysteines, as well as tricarboxylic acid cycle (TCA) intermediates such as succinate and fumarate^52–54^. Accumulation of HIF-1α invariably leads to the activation of hundreds of genes that carry a HRE on their promoter^2^.

UBXN7 is a cofactor protein that acts as a scaffold to assemble different polypeptides on the CRL2^VHL^ complex (Fig. 7). UBXN7 has four distinct domains and interacts with ubiquitinated HIF-1α (UBA domain), AAA + ATPase p97 (UBX domain), and neddylated CUL2 (UIM domain)^45^. The function of the UAS domain remains unknown. Previous studies have shown that overexpression of UBXN7 inhibits the CRL2^VHL^ complex leading to accumulation of HIF-1α; furthermore, using siRNA to inhibit UBXN7 expression causes a reduction in the HIF-1α protein level^36,43^. In this report we identify the adaptor UBXN7 protein as a ubiquitination substrate of the mitochondrial MUL1 E3 ligase. This is in accordance with previous results from a detailed proteomic analysis where MUL1 as well as UBXN7 proteins were found in complex with AAA + ATPase p97^55^. MUL1 has the ability to SUMOylate as well as to perform K48- or K63-linked ubiquitination on various substrates^21,24,26,29,34,40–42^. It has a unique topography located exclusively on the outer mitochondrial membrane^19^. MUL1 has a large and highly conserved inter membrane domain (IMD) that can potentially act as a stress sensor within the mitochondrial inter membrane space (IMS)^19,40^. MUL1’s ligase activity might also be modulated by mitochondrial stress, or through the availability of its partner E2 conjugation enzymes. MUL1 can form specific complexes with UbE2E3, UbE2D1, UbE2D2, and UbE2D3^56^. Each of these MUL1/E2 conjugating enzyme pairs has a unique specificity and targets substrates that are either cytoplasmic or associated to the outer mitochondrial membrane (Cilenti *et al*., unpublished). Furthermore, our previous studies identified MUL1 as a specific substrate of the mitochondrial Omi/HtrA2 serine protease^40,57^. Omi/HtrA2 protease is involved in protein quality control within the IMS and its inactivation in mice leads to the development of motor neuronal disease 2 (mnd2)^57,58^.

In this work we have identified two conserved ubiquitination sites on UBXN7, one in the UBA and the other in the UBX domains. In overexpression studies, we show ectopically expressed MUL1 is able to degrade endogenous UBXN7 but only if its ligase activity is intact. Furthermore, UBXN7 gene expression is not affected in the absence of MUL1, the level of UBXN7 mRNA is similar in HEK293 MUL1(+/+) and HEK293 MUL1(−/−) cells. HIF-1α protein is upregulated in HEK293 MUL1(−/−) cells that contain high levels of UBXN7 but is downregulated in HEK293 UBXN7(−/−) cells where the expression of UBXN7 is abolished. In either case, HIF-1α protein level closely mirrors that of UBXN7. Our data suggest that the UBXN7 cofactor plays a regulatory role as an inhibitor of the CRL2^VHL^ complex. High levels of UBXN7 also cause downregulation of some components of the CRL2^VHL^ complex including CUL2 and the AAA + ATPase p97. The protein level of Elongin B decreases, while Elongin C slightly increases but no detectable change was observed in the remaining components of CRL2^VHL^ complex (VHL and RBX1). Both CUL2 and AAA + ATPase p97 are necessary for the ubiquitination and extraction of HIF-1α from the CRL2^VHL^ complex, followed by its degradation by the proteasome. Changes in CUL2 and AAA + ATPase p97 that follow high levels of UBXN7 might be involved in the accumulation of HIF-1α. Alternatively, high levels of the adaptor UBXN7 protein without a concomitant increase of the other components might lead to the partial assembly of multiple CRL2^VHL^ complexes that are inactive and unable to function properly. This is supported by the fact that inhibition of neddylation in HEK293 MUL1(−/−) cells had no effect on HIF-1α protein level, implying the CRL2^VHL^ complex is inactive in the presence of high levels of UBXN7 protein.

We investigated the regulation of UBXN7 and HIF-1α in the presence or absence of MUL1 during hypoxia (1% oxygen). HEK293 MUL1(+/+) cells show the characteristic biphasic induction of HIF-1α as previously reported^59^. HEK293 MUL1(−/−) cells have a high basal level of HIF-1α that does not increase further with hypoxia, suggesting maximal expression of HIF-1α is attained in the absence of MUL1. The UBXN7 protein level correlates with the expression of HIF-1α under all conditions tested, suggesting a tight co-regulation of these two polypeptides. Both HEK293 MUL1(+/+) and HEK293 MUL1(−/−) cells show down-regulation of HIF-1α protein levels after prolonged hypoxia. This inhibition of HIF-1α protein levels, referred to as “the resolution phase,” seems to be independent of MUL1 but relies instead on the repressor element 1-silencing transcription factor (REST) as previously reported. REST binds directly to the HIF-1α promoter in a hypoxia-dependent manner and inhibits HIF-1α expression after prolonged hypoxia^60^. Hypoxia is the main regulator of glycolysis, as ATP production through oxidative phosphorylation (OXPHOS) is limited under low oxygen levels. HIF-1α is the key regulator of genes encoding enzymes involved in glucose metabolism that sustain ATP production during hypoxia. This is also consistent with the high levels of GLUT1 transporter found in the HEK293 MUL1(−/−) cells. Induction of glycolysis under aerobic conditions is the hallmark of many tumors, a phenomenon known as the Warburg effect^61–63^. HIF-1α independent glycolysis also exists, and can be achieved by the overexpression of the fasting/starvation-induced forkhead transcription factors FOXK1 and FOXK2^64^. Since inactivation of MUL1 leads to high levels of HIF-1α, we investigated the metabolic state of HEK293 MUL1(−/−) cells. We found that the HEK293 MUL1(−/−) cells, although they never experienced hypoxia, had their mitochondrial respiration compromised and switched their primary metabolic process to glycolysis. We assume it is the accumulation of HIF-1α alone, that causes the metabolic switch in HEK293 MUL1(−/−) cells. Since MUL1 is a E3 ligase with a broad function, it is also possible that besides the UBXN7 protein, other as yet uncharacterized substrate(s) might be involved in the induction of glycolysis in the HEK293 MUL1(−/−) cells.

It is becoming abundantly clear that aerobic glycolysis is paramount not only in cancer, but also in many biological processes involving proliferating cells as it provides a swift supply of ATP as well as the building blocks to support many cellular processes and cell growth^65–67^. This is the first report to show the existence of a new pathway where mitochondria, through MUL1, regulate HIF-1α protein levels. Our data clearly show this pathway is very important under normal conditions and could potentially be involved in the mitochondrial induction of aerobic glycolysis.

## Conclusion

We have identified a novel pathway that operates upstream of the CRL2^VHL^ ligase complex. This pathway involves the regulation of the UBXN7 cofactor by the mitochondrial MUL1 ligase. MUL1 ubiquitinates UBXN7 and targets it for degradation by the proteasome. This allows a tight regulation of the UBXN7 protein level that is kept low but is sufficient for the normal function of the CRL2^VHL^ complex. If UBXN7 is deregulated, as happens when MUL1 is inactive, the CRL2^VHL^ complex becomes inoperable leading to accumulation of HIF-1α and a metabolic shift from OXPHOS to glycolysis under aerobic conditions.

## Materials and Methods

### Cell culture and chemicals

HEK293 T-REx Flp-In HEK293 MUL1(+/+) and HEK293 MUL1(−/−) cells are described in^41^ and were kindly provided by Dr. Janos Steffen and Dr. Carla Koehler (Department of Biological Chemistry, David Geffen School of Medicine, Los Angeles, CA). HEK293 and HeLa cells were grown in Dulbecco’s modified Eagle’s medium (DMEM high glucose) supplemented with 10% fetal calf serum (Atlanta Biologicals), 2 mM L-glutamine, 1 mM sodium pyruvate, 50 units/ml penicillin, and 50 μg/ml streptomycin (ThermoFisher Scientific). MG132 (SIGMA) was dissolved in DMSO, stored at −20 °C, and used at the indicated concentrations. DMSO (0.1%) served as the vehicle, negative control.

### *In vitro* screening for Mulan/UbE2E3 ubiquitination substrates

The cytosolic RING finger domain of MUL1 (aa 259–352) was expressed in BL21(DE3) *E. coli* expression strain as a His-tagged protein using the pET-28a bacterial expression vector. After induction with IPTG, His-MUL1_259-352_ was purified on a Ni-NTA agarose column (Qiagen). The CDI HuProt™ microarrays (NextGen) were removed from −80 °C storage and placed at room temperature (RT) for 30 minutes in a sealed, desiccated container before opening to avoid formation of condensate. The arrays were rehydrated in a solution of 1 M urea, 0.5 M ethanolamine pH 7.5, 25% glycerol, 20 mM glutathione, and 1 mM DTT for 30 minutes at RT. The arrays were washed twice in PBST (PBS containing 0.1% Tween-20) and then blocked for 1 hour at RT in PBST containing 20 mM reduced glutathione, 1 mM DTT, 5% BSA, and 25% glycerol. Ubiquitination reaction was performed using TAMRA-labeled ubiquitin (Ub-TMR, Cat. No. SI270T), ubiquitin activating enzyme (UBE1, LifeSensors Cat. No. UB101), 100 nM UbE2E3 (LifeSensor Cat. No. UB213), and 100 nM His-MUL1_259-352_ for 60 minutes at RT. The ubiquitylation buffer also contained 20 mM HEPES pH 7.4, 150 mM NaCl, 0.5 mM DTT, 0.2 mM ATP, 2 mM MgCl_2_, and an ATP regeneration system consisting of creatine phosphate, creatine phosphokinase, and inorganic pyrophosphatase. A control array was treated with the same reaction mixture but without His-MUL1_259-352_. Arrays were washed with three changes PBST-1 (PBS containing 1% Tween-20), four changes of 0.2 micron-filtered deionized water, and then centrifugally dried (1000 × RPM for 5 minutes at RT). The arrays were scanned using a GenePix 4100 A Microarray Scanner (Molecular Devices, Inc.) with a 532 nm channel.

### His-UBXN7, His-UbE2E3 and GST-MUL1 protein expression and purification

UBXN7 (aa 1-489) and UbE2E3 (aa 1–207) recombinant cDNAs corresponding to the full-length proteins were cloned in frame into pET-28a bacteria expression vector (Novagen). pET-UBXN7 and pET-UbE2E3 plasmids DNA were transformed into BL21(DE3) *E. coli* expression strain and grown in 500 ml LB containing kanamycin 40 μg/ml at 37 °C until the OD at 600 nm reached 0.8 to 0.9. Expression of the recombinant proteins were induced with 2 mM IPTG at 25 °C for 4 hours. Bacteria pellets obtained after centrifugation were stored at −80 °C overnight followed by lysis in equilibration buffer (150 mM NaCl and 20 mM Tris-HCl pH 8) containing lysozyme at 100 μg/ml and protease inhibitors (SIGMA). Bacteria lysates were cleared by centrifugation, the supernatants were incubated with Ni-NTA beads and bound proteins eluted using equilibration buffer containing 20 mM imidazole. MUL1 full length cDNA (aa 1–352) was cloned in frame in pGEX-6P-1 gene fusion system. Bacteria growth, induction and expression of the recombinant protein was performed as described above. Fusion GST-MUL1 protein purification procedure was carried out using Glutathione Sepharose beads and eluted using 10 mM reduced glutathione in equilibration buffer (100 mM NaCl and 50 mM Tris-HCl pH 8). Aliquots were collected and the concentration and purity of the eluted protein was monitored by SDS-PAGE gel followed by Coomassie blue staining.

### *In vitro* ubiquitination assays

Purified His-UBXN7 (250 nM) was mixed together with purified GST-MUL1 (50 nM), purified His-UbE2E3 (250 nM), UBE1 (Life Sensors, 50 nM), and ubiquitin (Life Sensors, 50 µM) in reaction buffer (50 mM Tris-HCL pH 8, 5 mM MgCl_2_, 2 mM ATP and 1 mM β-ME). The ubiquitination reaction was allowed to proceed for 2 hours at 37 °C. The reaction was stopped by adding SDS sample buffer and by boiling the samples for 4 minutes. Ubiquitination reactions were resolved by SDS-PAGE followed by Western blot analysis using Ub specific antibodies.

### *In vitro* SUMOylation assay

Purified His-UBXN7 (250 nM) and purified GST-MUL1 (50 nM) were mixed together with the components of a SUMOylation kit (Enzo Life Sciences), and the assay was performed according to the manufacturer’s instructions. The reactions were resolved by SDS-PAGE followed by Western blot analysis using SUMO1 specific antibody.

### Ectopic expression of GFP-MUL1-WT, GFP-MUL1-H/A, or GFP-MUL1-C/A proteins

HEK293 cells were grown in six-well plates and transfected with GFP-C1 vector, GFP-MUL1-WT, GFP-MUL1-C/A, or GFP-MUL1-H/A (aa cysteine 339 or histidine 319 were replaced with alanine) plasmids using Lipofectamine 2000 (ThermoFisher Scientific) according to the manufacturer’s instructions. Twenty-four hours after transfection, cell extracts were prepared and the expression of the various endogenous or GFP-fusion proteins was monitored by SDS-PAGE and Western blot analysis.

### Confocal microscopy

HeLa cells were grown in 12-well plate on glass cover slips to 70% confluency and then transfected with 1 μg each of UBXN7-EGFP-N1 and MUL1-mRFP-C1 constructs using Lipofectamine 2000 Transfection reagent. After 24 hours, cells were treated with 2 μM MG132 or DMSO (control) for 4 h. The cover slips were then washed, fixed with 4% paraformaldehyde and mounted on a glass slide using Fluoromount-G as the mounting solution. Slides were observed using a Leica TCS SP5 II confocal laser-scanning microscope (Leica). The expression and stability of the UBXN7-GFP and RFP-MUL1 proteins were verified by Western blot analysis using anti-GFP and anti-RFP specific antibodies (SantaCruz).

### SDS-PAGE and western blot analysis

Control cells as well as cells treated with MG132 were lysed using a Triton X-100 based lysis buffer (1% Triton X-100, 10% glycerol, 150 mM NaCl, 20 mM Tris pH 7.5, 2 mM EDTA) in the presence of protease inhibitors tablets (ThermoFisher Scientific). Approximately 40 μg of whole cell extract was resuspended in SDS sample buffer, boiled for 5 minutes and the proteins resolved by SDS-PAGE. They were then transferred onto PVDF membranes (Genesee) using a semi-dry cell transfer blot (Bio-Rad) and placed in 4% nonfat dry milk in TBST buffer (25 mM Tris-HCl pH 8.0, 125 mM NaCl, 0.1% Tween 20) to block nonspecific binding of the membrane. The membranes were incubated with the indicated primary antibodies: MUL1 and UBXN7 rabbit polyclonal antibodies are homegrown and used at 1:5000 dilution, HIF-1α (Bioss Antibodies, 1:2000), p97 (SantaCruz, 1:3000), tubulin (SantaCruz, 1:2000), histone H3 (SantaCruz, 1:500), PHD2 (ProteinTech, 1:2000), GLUT1 (NovusBio, 1:1000), Elongin B (Aviva Systems Biology, 1:500), Elongin C (Aviva Systems Biology, 1:500), RBX1 (Aviva Systems Biology, 1:500), CUL2 (Thermo Fisher, 1:2000), Ub (Life Sensor, 1:1000), SUMO1 (ENZO, 1:1000), HA (Millipore, 1:2000), His-tag (ProteinTech, 1:500) and β-actin (SantaCruz, 1:3000). Secondary peroxidase-conjugated goat anti-rabbit or goat anti-mouse antibodies (Jackson ImmunoResearch) were used at 1:10,000 dilution; the membrane was visualized by enhanced chemiluminescence (ECL) (ThermoFisher Scientific).

### Subcellular fractionation

HEK293 MUL1(−/−) and HEK293 MUL1(+/+) cells were grown in 100 mm dishes. When the cells reached 95% confluence, they were detached using trypsin-EDTA (Invitrogen), washed twice with ice-cold PBS and processed for fractionation using the commercially available Nuclear and Cytoplasmic Extraction Kit (ThermoFisher Scientific) according to the manufacturer’s instructions. The fractions were analyzed by SDS-PAGE and Western blot using HIF-1α, tubulin and histone H3 specific antibodies.

### Hypoxic induction on HEK293 MUL1(+/+) and HEK293 MUL1(−/−)

Cells were cultured in complete DMEM media overnight and replaced with L-15 Medium (Leibovitz) before placing them inside a hypoxia incubator chamber (STEMCELL Technologies) using a gaseous mixture of 1% O_2_, 5% CO_2_ and 94% N_2_ for various times. Control cells were cultured under normoxic conditions (5% CO_2_) at 37 °C. Cells were harvested and washed with phosphate buffer saline. A fraction of the cells (1 × 10^5^) was used to monitor apoptosis. For this, cells were re-suspended in binding buffer, and AnnexinV-PE was added for 20 minutes at room temperature in the dark. Cells were then analyzed by flow cytometry using Cytoflex S (Beckman Coulter) as previously described^68,69^. The remaining cells were used to prepare cell extracts that were resolved by SDS-PAGE and protein levels of HIF-1α and UBXN7 were monitored.

### UBXN7 ubiquitination and mass spectrometric analysis

Duplicate 10 cm dishes of HEK293 cells were co-transfected with 5 μg of pcDNA-His-UBXN7 and 10 μg pcDNA-HA-ubiquitin (HA-Ub) plasmids. After 24 hours, one of the dishes was treated with 2 μM of proteasome inhibitor MG132 for 4 hours. Total cell lysates (2 mg) were mixed with Ni-NTA Agarose (Macerey-Nagel) gently rotating for 4 hours at 4 °C followed by 4x washes and bound proteins eluted in elution buffer (150 mM NaCl and 20 mM Tris-HCl) containing imidazole (200 mM). Ubiquitination analysis of the purified His-UBXN7 was performed by MtoZ Biolabs (Boston, MA).

### Site directed mutagenesis on UBXN7 protein to replace ubiquitinated residues

The two highly conserved lysine (K) amino acid residues (K14 and K412) identified as the only ubiquitination sites on UBXN7 were replaced with arginine (R) to create three mutant UBXN7 proteins; two of them carry a single (K14 or K412) substitution and the third is a double mutant (both K14 and K412). Specific primers were designed where the codon for K14 or K412 was switched to an R. We used the Q5 site-directed mutagenesis kit (New England BioLabs) and followed the instruction’s manual.

### Generation of UBXN7 knockout cells using CRISP/Cas9 gene editing

To ablate UBXN7 expression, the target sequence TCTGTGTTGTTGTTCGGCGGCGG in the exon 1 was selected using the CRISPOR program (http://crispor.tefor.net) and cloned into the BbsI site of pSpCas9(BB)-2A-GFP (PX458) vector (Addgene) as previously described^70^. The resulting vector and an empty PX458 vector were transfected into HEK293 cells and 48 hours later, single GFP-positive cells were sorted into 96 well plates using a BD FACS ARIA II sorter. Clones were expanded and tested for lack of UBXN7 protein expression. In addition, genomic DNA was isolated for PCR amplification of the region surrounding exon 1 including the ATG start site of UBXN7 and subsequently analyzed using ALT-R genome editing detection kit (IDT). One clone each from the HEK293 UBXN7(−/−) and control HEK293 UBXN7(+/+) were selected for further experiments.

### Mitochondrial stress assay

Mitochondrial stress assay was performed using an XF^e^24 Extracellular Flux Analyzer (Agilent) following the workflow provided by the manufacturer’s instructions. Briefly, for Oxygen Consumption Rate (OCR) and Extracellular Acidification Rate (ECAR) measurements, HEK293 MUL1(−/−) and HEK293 MUL1(+/+) cells were seeded in triplicates on poly-lysine D-coated XF24 microplates at a density of 80,000 cells per well in assay medium (XF DMEM medium pH 7.4 supplemented with 10 mM glucose, 2 mM glutamine, 1 mM pyruvate), followed by incubation at 37 °C in a non-CO_2_ incubator for 60 minutes. Three baseline measurements were recorded before the injection of the following compounds: Oligomycin in port A (56 µl) at 1.0 μM, FCCP in port B (62 μl) at 1.0 μM, and Rotenone/Antimycin in port C (69 μl) at 0.5 μM. Data analysis was performed using Cell Mito Stress Test Report Generators software.

### Real-time ATP rate assay

For ATP production rate assay an Agilent Seahorse XF Real-Time ATP Rate Assay Kit was used and HEK293 MUL1(−/−) and HEK293 MUL1(+/+) cells were plated as described above and ATP measurements were recorded followed by Oligomycin injection in port A (56 µl) at 1.5 μM final concentration, and Rotenone/Antimycin injection in port B (62 μl) at 0.5 μM final concentration. Data analysis was performed using Report Generators software for Real-Time ATP Rate Assay.

### Glycolytic rate assay

For the Glycolytic rate assay seahorse XF Glycolytic Rate Assay kit was used. Rotenone/Antimycin at 0.5 μM each was used in port A and the glycolysis inhibitor, 2-deoxy-D-glucose (2-DG), was subsequently injected in port B (62 µl) at a concentration of 50 mM. To asses glycolysis, three measurements were recorded after the addition of each compound by a 3-2-3 mix/measurement cycle. As a negative control, three wells with no cells were used both in the OCR and ECAR analysis. Data analysis was performed using Report Generators software for the Glycolytic Rate Assay.

### Statistical analysis

All quantitative data are expressed as mean ± SD or ± SEM of three or four independent experiments. Following Western blot analysis, the optical densities of blot bands were determined using ImageJ software. Protein/β-actin ratios were obtained from the densitometry data, and the differences among groups were analyzed by one tailed Student’s *t* test. A value of *p* < 0.05 was considered significant. All Seahorse data were analyzed using Report Generators software that automatically calculate and report the assay parameters of the Agilent Seahorse XF^e^24 (Agilent) specific for each assay (Cell Mito Stress, ATP rate and Glycolysis assay).

## Supplementary information

Supplementary figure 1.

Supplementaryinfomation.
