# Supplementary material for: Mitochondrial MUL1 E3 ubiquitin ligase regulates Hypoxia Inducible Factor (HIF-1α) and metabolic reprogramming by modulating the UBXN7 cofactor protein
Source: Sci Rep. 2020 Jan 31;10:1609. doi: 10.1038/s41598-020-58484-8 (PMC6994496; doi:10.1038/s41598-020-58484-8)
Supplement: Supplementary file 1 — Supplementary figure 1. [file 41598_2020_58484_MOESM1_ESM.pdf]

Mitochondrial MUL1 E3 ubiquitin ligase regulates Hypoxia Inducible Factor (HIF-1α) and metabolic reprogramming by modulating the UBXN7 cofactor protein

Lucia Cilenti [1#], Jacopo Di Gregorio [1#], Camilla T. Ambivero [1], Thomas Andl [1], Ronglih Liao [2], and Antonis S. Zervos [1*]

[1] Burnett School of Biomedical Sciences, University of Central Florida College of Medicine 12722 Research Parkway Orlando, FL 32826

[2] Stanford Cardiovascular Institute, Stanford University School of Medicine, 1651 Page Mill Road, Palo Alto, 94043

#These authors contributed equally to this work

*Correspondence and requests for materials should be addressed to A.S.Z.
(email: Antonis.Zervos@ucf.edu)

Supplementary Materials

Quantitative real-time PCR

Total cellular RNA was isolated from HEK293 MUL1(+/+) and HEK293 MUL1(-/-) cells using the RNAeasy Mini Kit (Qiagen). First-strand cDNA was generated using the QuantiTect Reverse Transcription Kit (Qiagen) according to the manufacturer's protocol. Briefly, 500 ng of RNA was reverse transcribed in a 20 μl volume and after heat-inactivation diluted with 80 μl of water. For the qRT-PCR, 2 μl of the diluted RT reaction was used in a final volume of 10 μl. qRT-PCR was carried out with the Rotor-Gene SYBR Green PCR Kit (Qiagen #204076) using the following Qiagen QuantiTect primers: VEGF-A (Qiagen, VEGF-A 6 SG QT01682072), EIF3D (Qiagen, EIF3D 1 SG, QT00010829), and β-actin (Qiagen, ACTB 1 SG QT00095431). The UBXN7 primers used were: 5' CAGACAAAACAGGATAGCCGCTCA, 3' ATTTACATCTATCCCCTCCACTAC. The qPCR reactions were run in a Rotor-Gene Q instrument (Qiagen) for 40 cycles (95°C 7 seconds and 60°C 20 seconds) after an initial denaturation step of 5 minutes. All reactions were performed in triplicates. Cycle threshold (Ct) values were obtained using a fixed threshold setting and values were normalized with the EIF3D Ct data. Data were analyzed with the $2^{-\Delta\Delta Ct}$ Livak-method to determine changes in UBXN7 and VEGF-A expression, while β-actin served as a control.

Fig. S1

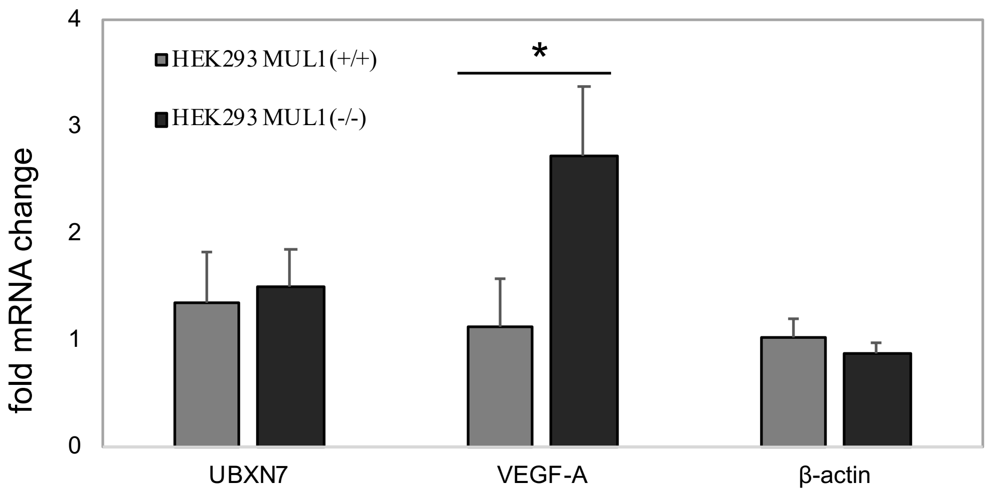

Supplementary Figure S1. Quantitative real-time PCR analysis of UBXN7 and VEGF-A in HEK293 MUL1(+/+) and HEK293 MUL1(-/-) cells. No significant changes in UBXN7 mRNA level was observed in HEK293 MUL1(-/-) compared to HEK293 MUL1(+/+) cells. As a positive control, VEGF-A (an HIF-1α target gene) was used which shows a significant (*p<0.0006, Student's t test) 2.4-fold induction of expression in the absence of MUL1. β-actin was used as a control for a gene unaffected by changes in MUL1 expression. EIF3D served as the normalizer in these experiments.
