# Supplementary material for: Mitochondrial MUL1 E3 ubiquitin ligase regulates Hypoxia Inducible Factor (HIF-1α) and metabolic reprogramming by modulating the UBXN7 cofactor protein
Source: Sci Rep. 2020 Jan 31;10:1609. doi: 10.1038/s41598-020-58484-8 (PMC6994496; doi:10.1038/s41598-020-58484-8)
Supplement: Supplementary file 2 — Supplementaryinfomation. [file 41598_2020_58484_MOESM2_ESM.pdf]

Mitochondrial MUL1 E3 ubiquitin ligase regulates Hypoxia Inducible Factor (HIF-1α) and metabolic reprogramming by modulating the UBXN7 cofactor protein

Lucia Cilenti [1#], Jacopo Di Gregorio [1#], Camilla T. Ambivero [1], Thomas Andl [1], Ronglih Liao [2], and Antonis S. Zervos[1 *]

Supplementary information

Original, uncropped Western Blots:

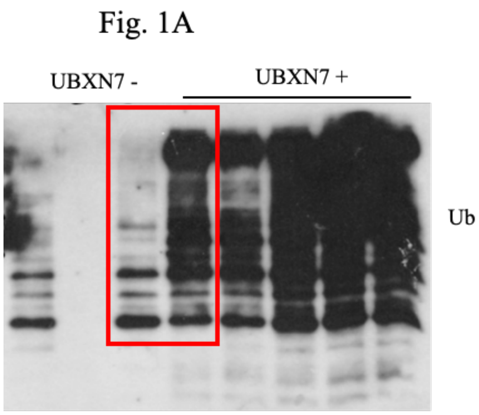

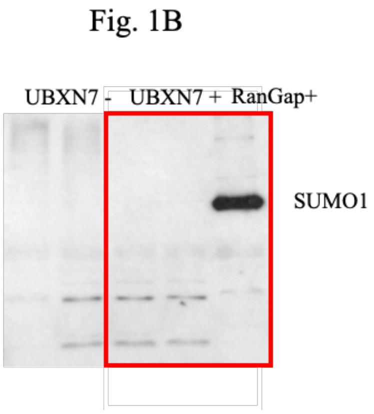

Fig. 1C

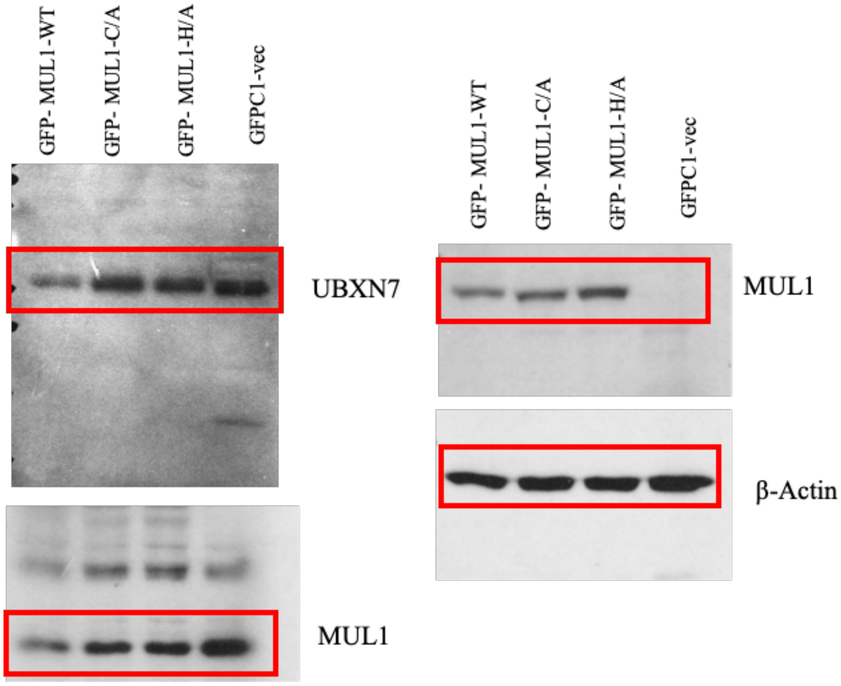

Original Western blots related to Figure 1

Fig. 2E

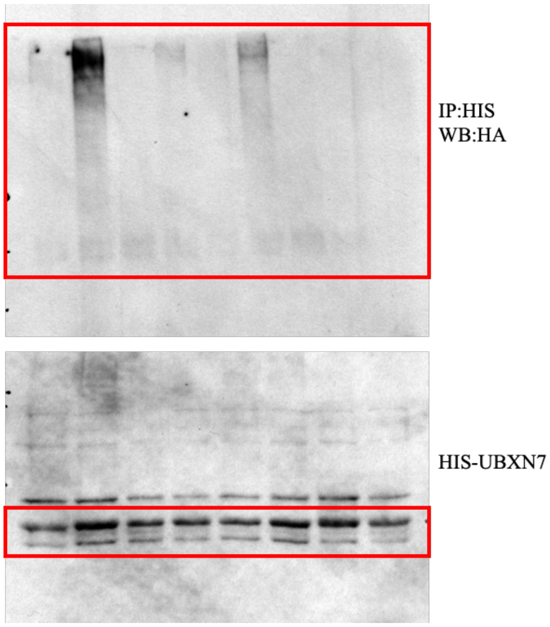

Fig. 2F

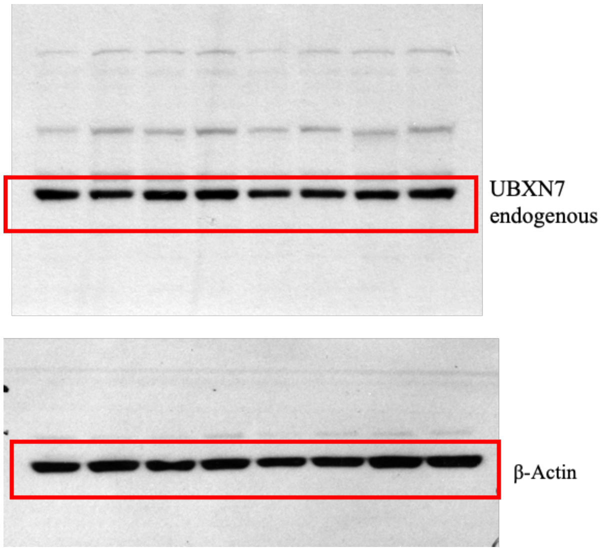

Original Western blots related to Figure 2

Fig. 3A

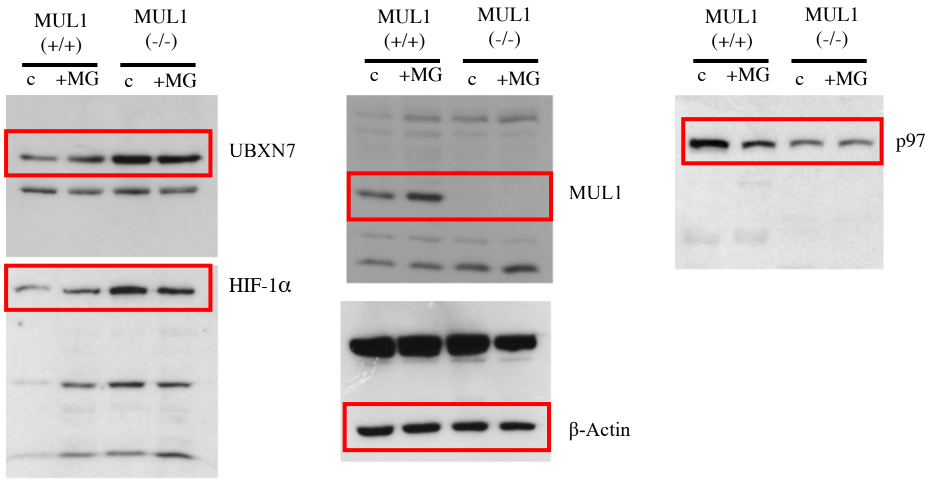

Fig 3C

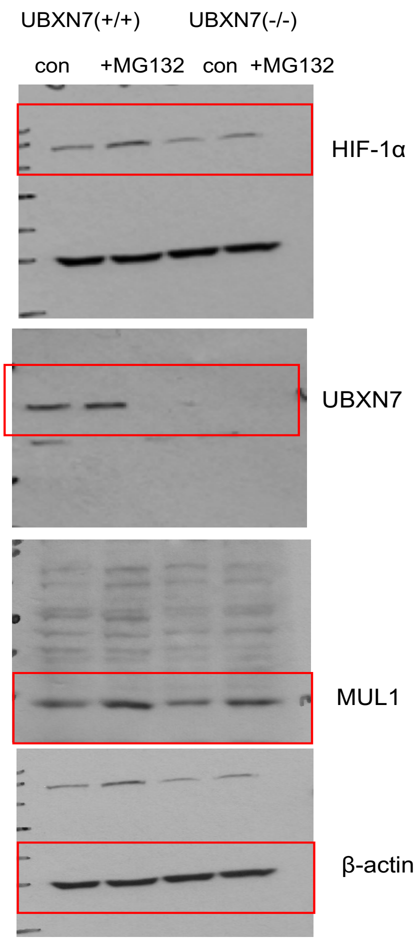

Fig3E

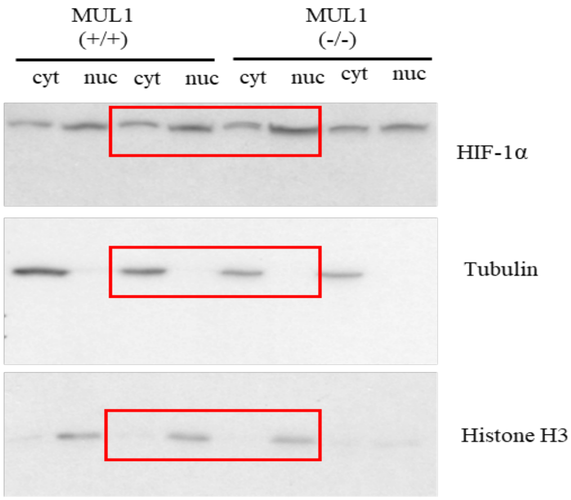

Fig. 3G

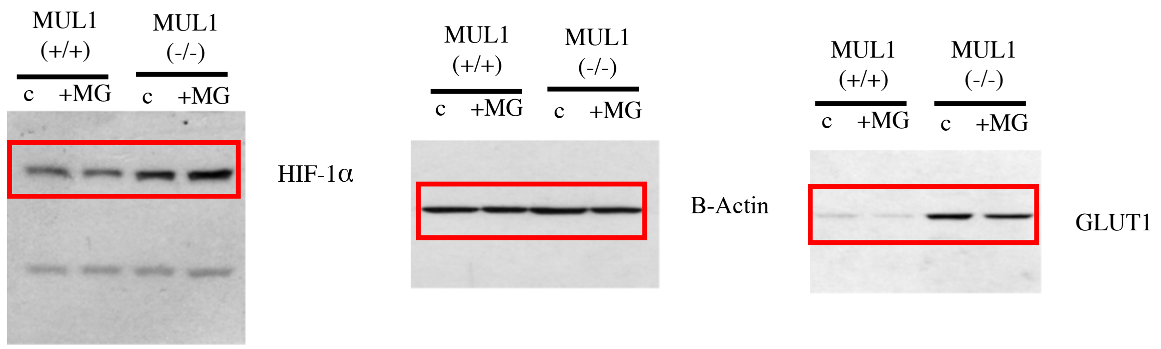

Original Western blots related to Figure 3

Fig. 4A

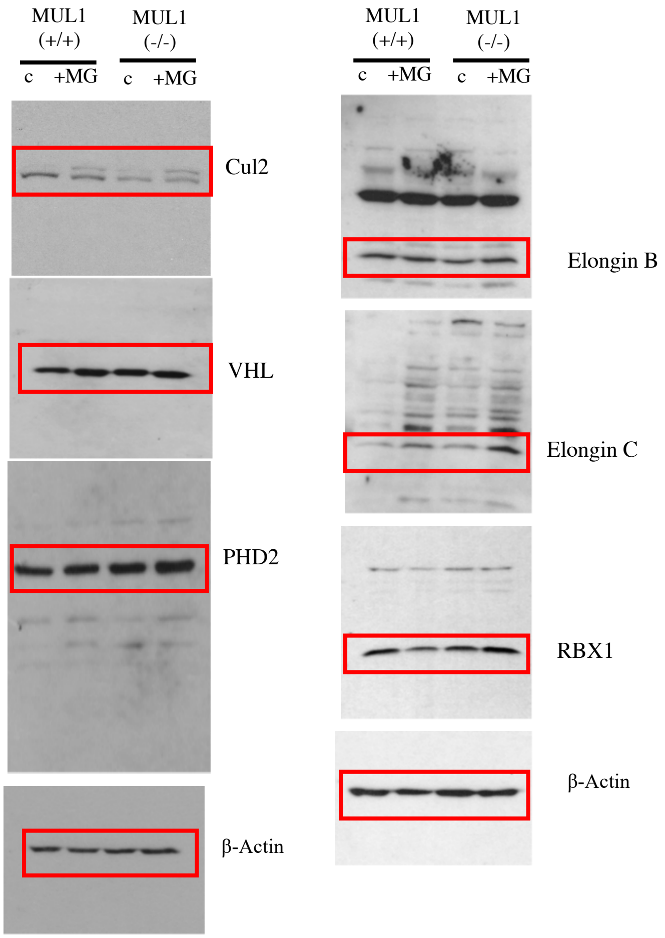
MUL1 (+/+)
MUL1 (-/-)
c
+MG
c
+MG
Cul2
VHL
PHD2
β-Actin
MUL1 (+/+)
MUL1 (-/-)
c
+MG
c
+MG
Elongin B
Elongin C
RBX1
β-Actin

Fig 4C

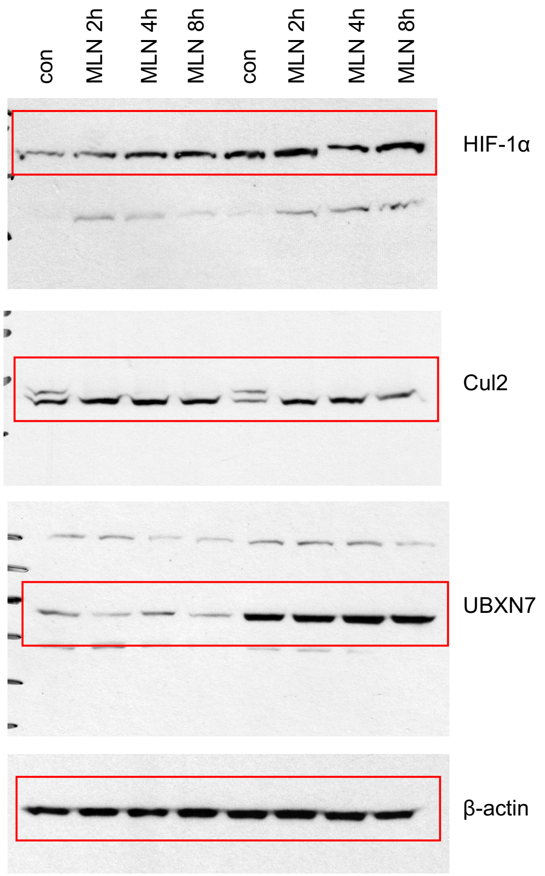

Original Western blots related to Figure 4

Fig. 5A

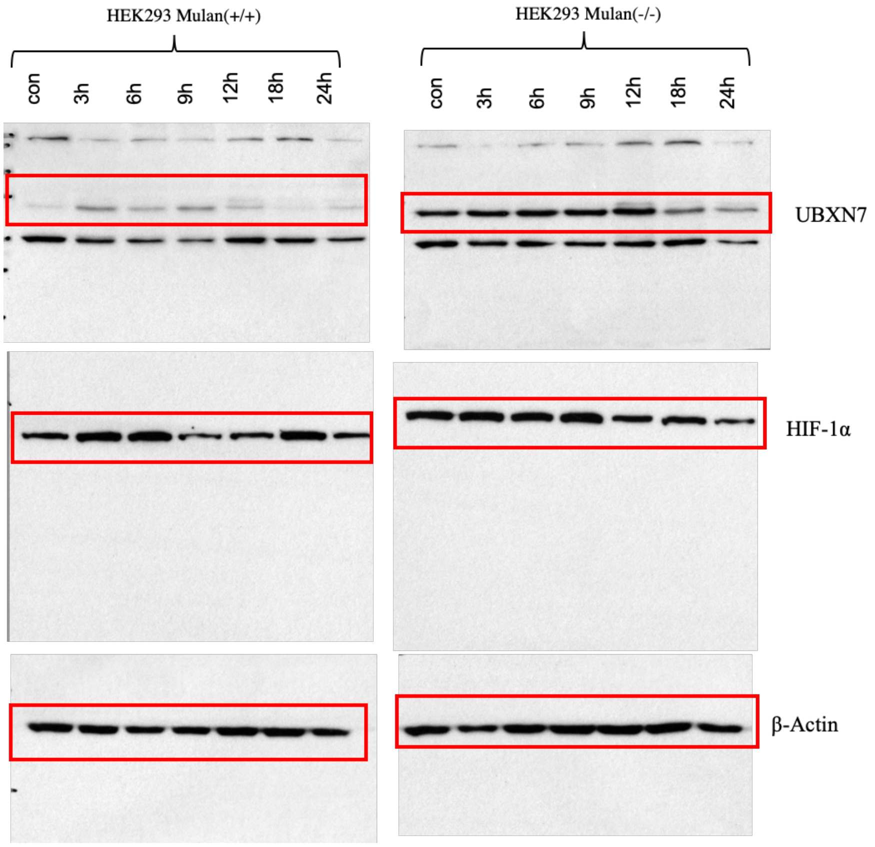

Original Western blots related to Figure 5
